# Quantitative Determination of three Angiotensin-II-receptor Antagonists in Presence of Hydrochlorothiazide by RP-HPLC in their Tablet Preparations 

**Published:** 2013

**Authors:** Hany Mohammed Hafez, Abdullah Ahmed Elshanawane, Lobna Mohammed Abdelaziz, Magda Mohammed Kamal

**Affiliations:** Medicinal Chemistry Department Faculty of Pharmacy, Zagazig University, Zagazig, Egypt. Chemistry, Nancy University, France, QC Manager, EIPICO, 10^*th *^Ramadan, Egypt.

**Keywords:** RP-HPLC, Angiotensin-II-receptor, Hydrochlorothiazide

## Abstract

Losartan potassium, Valsartan , Telmisartan and Irbesartan are angiotensin-II-receptor antagonists (ARA II) group which used in treatment of hypertension alone or in combination with other drugs mainly Hydrochlorothiazide. RP- HPLC method was developed for the assay of three angiotensin-II-receptor antagonists (ARA-IIs) in presence of Hydrochlorothiazide. The method was performed by reversed phase high performance liquid chromatography using a mobile phase which is consisted of 0.025 M potassium dihydrogen phosphate (pH 6.0): acetonitrile = 65:35% with detection at 220 nm on an ACE C18 column (250 mm × 4.6 mm, 5 μm) at flow rate 1.5 mL/min in an isocratic manner. The proposed method was validated according to ICH guidline in terms of linearity, accuracy, precision , robustness, limit of detection and limit of quantitation.

## Introduction

Angiotensin antagonists are the first major innovation in essential hypertension management as a first-line treatment. Angiotensin II receptor antagonists (ARA II) have been developed to specifically and selectively block the AT1 receptor of the rennin angiotensin system by displacing angiotensin II from it (1). Losartan potassium, Telmisartan and Irbesartan are highly selective, non-peptide angiotensin-II receptor antagonist s (ARA-II). They are effective agents for the treatment of hypertension and heart failure either alone or together with diuretics or recently with other antihypertensive drugs (2). So, it is necessary to develop a validated analytical method for assay of ARAII in combination with hydrochlorothiazide in its pharmaceutical preparations. Literature review revealed that USP described RP-HPLC methods for assay of Losartan potassium and ion pair HPLC for Irbesartan and Telmisartan. It described a gradient RP-HPLC method for assay of Losartan potassium in combination with hydrochlorothiazide and a gradient ion pair HPLC for Irbesartan in combination with hydrochlorothiazide (3). BP described a potentiometric titration for assay of Losartan potassium, Irbesartan and Telmisartan (4). Some methods have been published for simultaneous determination of studied ARA-II-drugs separately in combination with hydrochlorothiazide in its pharmaceutical preparations (5-12). EIPICO Company described a RP-HPLC method for analysis of Losartan potassium and hydrochlorothiazide (50/12.5 mg) tablets. Analysis was performed on a Hypersil C18 column using mobile phase consisted of a mixture of acetonitrile and phosphate buffer (pH 3.5; 0.05 M) (50:50% v/v) and detector was set at 220 nm (13) other methods have been reported for determination of only ARAII drugs (14-16). Only one method has been established to assay ARAII and hydrochlorothiazide by electrophoresis (17) but this technique is less available in pharmaceutical companies than HPLC-UV and more expensive so, it is preferable that the developed method is HPLC-UV, the most spreadable apparatus in pharmaceutical companies. Our recent method is characterized by a simplicity, accuracy, preciseness and sensitivity. 

**Figure 1:**
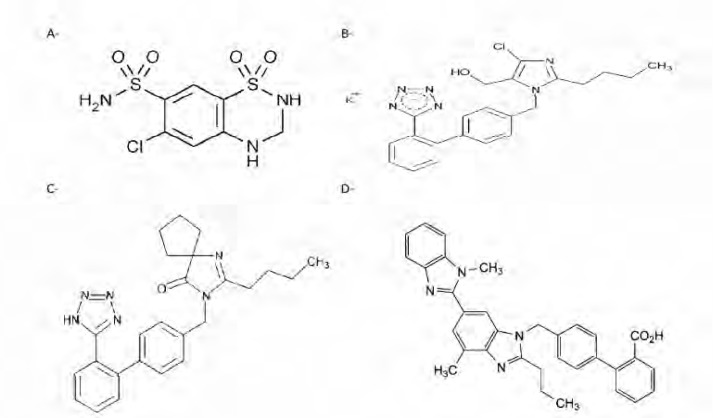
Structures of a- Hydrochlorothiazide b- Losartan potassium c- Irbesartan d- Telmisartan

Hydrochlorothiazide is 2H -1, 2, 4-Benzothiadiazine-7-sulfonamide, 6-chloro-3, 4-dihydro 1, 1-dioxide; Hydrochlorothiazide is the most famous thiazide diuretics. Losartan potassium is 2-butyl-4-chloro-1-[[2’-(1H-tetrazol-5-yl)[1,1’-biphenyl]-4-yl]methyl]-1H-imidazole-5-methanol monopotassium salt, is the first member of a new class of non-peptide angiotensin II receptor antagonist .Irbesartan is 2-butyl-3-[p-(o-1H-tetrazol-5-ylphenyl) benzyl]-1, 3-diazaspiro (4.4) non-1-en-4-one. It is an orally active specific angiotensin II receptor antagonist used, as a hypotensive agent does not require biotransformation into an active form. Valsartan is N-(1-oxopentyl)-N-[[2-(1H-tetrazol-5-yl) [1, 1-biphenyl]-4-yl] methyl]-l-valine (Figure 1).

## Experimental

*Instrumentation*

The HPLC system used was a computer based Agilent 1200 series instrument comprising of a quaternary pump, a UV detector and Auto sampler (injector). The system equipped by Agilent chemistation PC program. 

*Chemicals and reagents*

All reagents used were of analytical grade or HPLC grade. Potassium dihydrogen phosphate, orthophosphoric acid and Sodium hydroxide (NaoH) were supplied by (Merck, Darmstadt, Germany), Acetonitrile and Methanol HPLC grade were supplied by (Fischer scientific, U.K.) and Distilled water. 

(Note: The water used in all the experiments was obtained from Milli-RO and Milli-Q systems (Millipore, Bedford, MA). 

*Irbesartan, Losartan potassium, Telmisartan *and *Hydrochlorothiazide *working standard powders were kindly supplied by Egyptian international pharmaceutical industries company (EIPICO) (10th Ramadan, Egypt), and were used without further purification.

*Pharmaceutical preparation*

*X-tension plus *tablets October pharma/EPCP (Egypt) contain (150 mg Irbesartan + 12.5 mg Hydrochlorothiazide) per tablet B.NO: E0230311. *Losazide *tablets (EIPICO, Egypt) contain (50 mg Losartan potassium + 12.5 mg Hydrochlorothiazide) per tablet B.NO:1002445. *Micardis plus *tablets (Boehringer Ingelheim Company, Germany) contain (80 mg Telmisartan + 12.5 mg Hydrochlorothiazide) per tablet B.NO:908577.

*Chromatographic condition*

The chromatographic separations were performed on BDS Hypersil C18 (250 mm, 4.6, 5μm i.d ) at column temperature 40 °C, using a mobile phases consisting of a mixture of potassium dihydrogen phosphate buffer (pH 6.0, 0.025M) and acetonitrile (65:35, V/V) and pH was adjusted to 6.0 with 1 M NaoH. The injection volume was injected 50 μL at a flow rate of 1.4 mL/min and detection was performed at 220 nm using a UV detector. Mobile phase was filtered through a 0.45 μL Nylon membrane under vacuum and degassed by ultrasonication before usage.

*Preparation of stock standard solutions*

Stock standard solutions containing (1.25, 1.5, 0.5, 0.8 mg/mL) of Hydrochlorothiazide, Irbesartan, Losartan potassium, Telmisartan respectively were prepared by dissolving (12.5, 150, 50, 80 mg) of each in methanol in 100 mL volumetric flask respectively. It was then sonicated for 15 min and the final volume of solutions was made up to 100 mL with methanol to get stock standard solutions.

*Preparation of calibration plot (working standard solutions)*

To construct calibration plots, The stock standard solutions were diluted with the mobile phase to prepare working solutions in the concentration ranges (2.5-15, 30-180,10-60,16-96 μL/mL) for Hydrochlorothiazide, Irbesartan, Losartan potassium and Telmisartan respectively. Each solution (n = 5) was injected in triplicate and chromatographed under the mentioned conditions above. Linear relationships were obtained when average drug standard peak area were plotted against the corresponding concentrations for each drug. Regression equation was computed.

*Sample preparation*

A composite of ten *X-tension plus *tablet, *Losazide *tablet and *Micardis plus *tablet were prepared by grinding them to a fine, uniform size powder, triturated using mortar and pestle.

**Figure 2:**
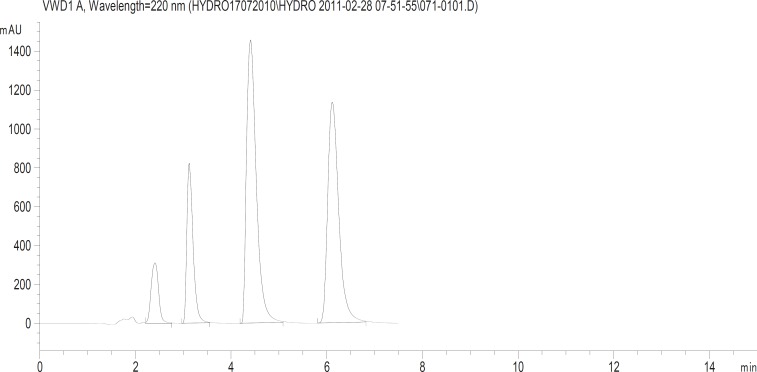
Typical HPLC chromatograms obtained from 50 μL injections of Hydrochlorothiazide, Losartan potassium, Irbesartan and Telmisartan respectively under optimized chromatographic conditions

After calculating the average tablet weight, amounts of powder equivalent to (2.5, 150, 50 and 80 mg) for Hydrochlorothiazide, Irbesartan, Losartan potassium and Telmisartan respectively of each type of tablets were accurately weighed and transferred separately to 100 mL volumetric flasks respectively. Solutions were sonicated for 15 min and the solutions were then filtered through 0.45 mL Nylon membrane filters (Millipore, Milford, MA, USA). Aliquots of appropriate volume (10 mL) were transferred to 100 mL calibrated flasks and diluted to volume with mobile phase to furnish the mentioned concentration above. The diluted solutions were analyzed under optimized chromatographic conditions and chromatogram is depicted in (Figure 2).

## Results

*Method validation*

*Specificity*

**Figure 3:**
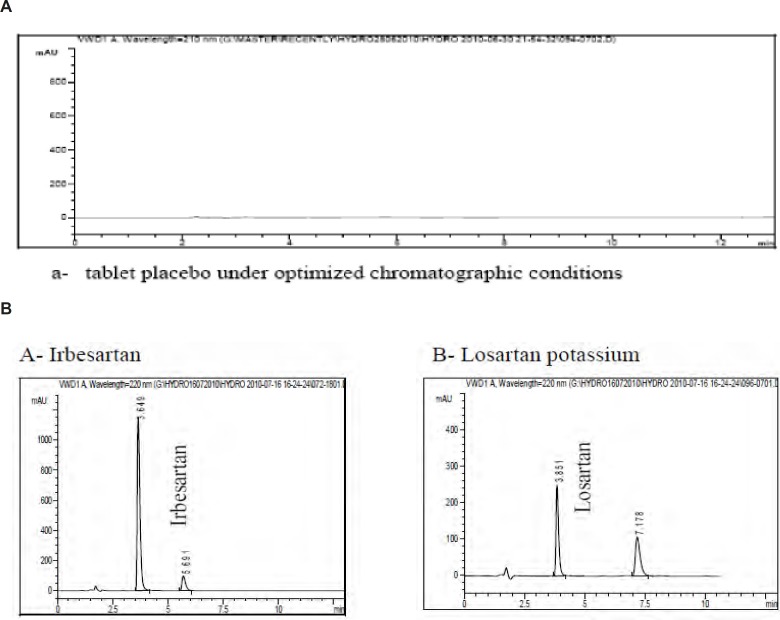
Typical HPLC chromatograms obtained from 50 μL injections of a- tablet placebo under optimized chromatographic conditions **b- **Irbesartan and Losartan potassium respectively obtained from stress studies involving acid, base and heat as well as analysis of samples stored under ICH stability conditions under optimized chromatographic conditions

Specificity of the method was evaluated by assessing whether excipients present in the pharmaceutical formulations interfered with the analysis or not (18). A placebo for each tablet was prepared by mixing the respective excipients and solutions were prepared by following the procedure described in the section of sample preparation. The commonly used tablet excipients did not interfere with the method. The diluent chromatogram in (Figure 3) shows that the tablet diluent has negligible contribution after the void volume at the method detection wavelength of 220 nm.

The method were also evaluated by assessing whether degradation products present in the pharmaceutical formulations interfered with the analysis, obtained from stress studies involving acid, base, peroxide and heat as well as analysis of samples stored under ICH stability conditions. Chromatograms are also shown in (Figure 3) to demonstrate method specificity.

*Linearity and range*

The linearity of the method was evaluated by analyzing different concentration of the drugs. According to ICH recommendations (18) at least five concentrations must be used. In this study five Concentrations were chosen, in the ranges (2.5-15, 30-180, 10-60 and 16-96 μL/mL) corresponding levels of 20-120% w/w of the nominal analytical concentration for Hydrochlorothiazide, Irbesartan, Losartan potassium and Telmisartan respectively. The linearity of peak area responses versus concentrations was demonstrated by linear least square regression analysis. The linear regression equations were {Y = 223.09 X + 4.000 (r= 0.9999), Y = 122.54 X + 0.8806(r= 1.0), Y = 138.95 X + 9.9936(r= 1.0), Y = 197.86 X + 120.99 (r= 0.9997)} for Hydrochlorothiazide, Irbesartan, Losartan potassium and Telmisartan respectively. Where **Y **is the peak area of standard solution and X is the drug concentration. 

*Precision*

The precision of the assay was investigated by measurement of both repeatability and Intermediate precision.

*Repeatability*

Repeatability was investigated by injecting 6 determinations at 100% of the test concentration percentage RSD were calculated in Table1.

**Table 1:** Repeatability of Hydrochlorothiazide, Irbesartan, Losartan potassium and Telmisartan respectively

**Drug name**	**Average μg/mL**	**Average %**	**RSD**
Hydrochlorothiazide	12.53	100.26	0.32%
Irbesartan	150.33	100.22	0.38%
Losartan potassium	50.09	100.17	0.38%
Telmisartan	79.35	99.19	0.39%

*Intermediate precision*

In the inter-day studies, standard and sample solutions prepared as described above, were analyzed in triplicate on three consecutive days at 100% of the test concentration and percentage RSD were calculated (Table 2).

**Table 2:** Intermediate precision of Hydrochlorothiazide, Irbesartan, Losartan potassium and Telmisartan respectively

**Drug name**	**1** ^st^ ** day** **μg/mL**	**2** ^n^ **d day** **μg/mL**	**3** ^rd^ ** day** **μg/mL**	**pooled average**	**pooled average %**	**RSD**
Hydrochlorothiazide	12.53	12.81	13.01	12.78	102.2	1.88%
Irbesartan	150.33	150.1	150.9	150.8	100.5	0.25%
Losartan potassium	50.08	50.38	50.38	50.28	100.57	0.34%
Telmisartan	80.03	79.35	79.3	79.56	99.45	0.51%

*Accuracy*

Accuracy was assessed using 9 determinations over 3 concentration levels covering the specified range (80,100 and 120%). Accuracy was reported as percent recovery by the assay of known added amount of analyte in the sample (Table 3).

**Table 3:** Recovery results for standard solution plus excipients for Hydrochlorothiazide, Irbesartan, Losartan potassium and Telmisartan respectively

**Drug name**	**Recovery at 80% conc. (%)**	**Recovery at 100% conc. (%)**	**Recovery at 120% conc. (%)**	**Average Recovery (%)**	**RSD**
Hydrochlorothiazide	99.10	99.18	99.34	99.21	0.12%
Irbesartan	100.62	99.80	99.87	100.10	0.45%
Losartan potassium	100.62	99.78	99.94	100.11	0.45%
Telmisartan	101.25	98.82	99.03	99.70	1.35%

*Limits of detection and limits of quantitation*

According to the ICH recommendations (18), determination of limits of detection and quantitation was based on the standard deviation of the y-intercepts of regression lines and the slope of the calibration plots (Table 4).

**Table 4:** Calibration data was resulted from method validation of Hydrochlorothiazide (HCTZ), Irbesartan, Losartan potassium and Telmisartan respectively

**Item**	**HCTZ**	**Irbesartan**	**Losartan**	**Telmisartan**
Linear range (μg/mL)	2.5-15	30-180	10-60	16-96
Detection limit (μg/mL)	0.005	0.08	0.007	0.04
Quantitation limit (μg/.mL)	0.017	0.24	0.021	0.13
Regression data				
N	5	5	5	5
Slope (b)	258.73	148.08	137.13	228.47
Standard deviation of the slope	0.83	0.46	0.55	0.88
Intercept (a)	2.01	15.22	46.76	96.19
Standard deviation of the intercept	2.11	2.26	9.84	9.23
Correlation coefficient ®	0.9998	1.0	1.0	1.0
Standard error of regression	0.08	0.44	0.14	0.25

(Y = a + bC, where C is the concentration of the compound (μg/mL) and Y is the drug peak area).

*Robustness*

Robustness of an analytical procedure is a measure of its capacity to remain unaffected by small variations in method parameters and provides an indication of its reliability during normal usage (18). Robustness was tested by studying the effect of changing mobile phase pH by ± 0.1, the amount of acetonitrile in the mobile phase by ± 2%, temperature ± 2°C different column and flow rate ± 0.05 mL/min had no significant effect on the chromatographic resolution of the method.

*Stability of analytical solution*

Also as part of evaluation of robustness, solution stability was evaluated by monitoring the peak area response. Standard stock solutions in methanol were analyzed right after its preparation 1, 2 and 3 days after at 5 °C and for a day at room temperature. The change in standard solution peak area response over 3 days was (1.67, 0.35, 0.39 and 0.53%) for Hydrochlorothiazide, Irbesartan, Losartan potassium and Telmisartan respectively. Their solutions were found to be stable for 3 days at 5 °C and for a day at room temperature at least. 

## Discussion

*Optimization of chromatographic condition*

To establish and validate an accurate method for analysis of these drugs in pharmaceutical formulations, preliminary tests were performed with the objective of selecting optimum conditions. To reach our goal, BDS Hypersil column (25 cm), Hypersil cyano (CN) and Hypersil CPS columns were tried for simultaneous determination of the drugs. BDS Hypersil column (25 cm) is the most hydrophobic stationary phase so, it gave good separation between these drugs but drugs eluted very slowly especially Telmisartan. The effect of mobile phase composition were also studied (a) aqueous phase *e.g*. ammonium acetate buffer and phosphate buffer (b) organic modifier *e.g*. acetonitrile and methanol (c) pH of aqueous phase *e.g. *3.5, 6.0.

Changing pH of mobile phase from 3.5 to 6.0 affects on eluting of Telmisartan to be more faster (10 min) because It is ionizable compound containing carboxylic group (COOH), pH of mobile phase greater than pKa of Telmisartan (4.45) by more one unit and At pH’s above the pKa of the analyte, the acidic analyte carries a negative charge and behaves as an extremely polar molecule and also eluting of losartan potassium (pKa = 3.1) and irbesartan (pKa = 4.7) (due to presence of tetrazole ring which is also a weak acidic group similar to a carboxcylic acid group ) were affected to be more faster by elevating pH to 6.0 but with lesser extent only 4-5 min (19, 20). The optimum wavelength for detection was 220 nm at which much better detector responses for four drugs were obtained. The best resolution with reasonable retention time was obtained at 65% phosphate buffer pH 6.0 and 35% acetonitrile as organic modifier. A major reason for using a concentration of 25 mM was achieving maximum sensitivity of UV detection at low wavelengths.

*Application on pharmaceutical preparation*

The proposed methods were successfully used to determine Irbesartan, Losartan potassium, Telmisartan and Valsartan respectively in their dosage forms in presence of Hydrochlorothiazide *e.g*. X-tension plus tablets, Losazide tablets and Micardis plus tablets respectively. Five replicate determinations were performed. Satisfactory results were obtained for each compound in good agreement with label claims (Table 5 and 6). The results obtained were compared statistically with those from published methods (5, 11 and 13) by using Student’s t-test and the variance ratio F-test. The results showed that the t and F-values were smaller than the critical values. So, there were no significant differences between the results obtained from this method and published methods (Table 7).

**Table 5:** Results from determination of Irbesartan (IRB), Losartan potassium (LOS) and Telmisartan (TEL) in presence Hydrochlorothiazide (HCTZ) respectively in their dosage forms by proposed method

**Product name**	**X-tension** **plus tablets**		**Losazide tablets**		**Micardis plus tablets**	
**Drug name**	**IRB** **(%)**	**HCTZ** **(%)**	**LOS** **(%)**	**HCTZ** **(%)**	**TEL** **(%)**	**HCTZ** **(%)**
Test 1	97.97	97.9	99.91	99.39	99.78	99.71
Test 2	99.99	98.24	100.8	99.49	99.4	99.17
Test 3	99.52	98.96	99.88	100.1	99.64	99.3
Test 4	99.38	99.05	101.1	99.65	99.27	99.02
Test 5	99.45	99.66	100.1	98.72	99.44	99.07
SD	0.76	0.7	0.56	0.50	0.20	0.280
Average	99.26	98.76	100.35	99.47	99.51	99.25
R.S.D	0.766	0.71	0.56	0.50	0.20	0.28

**Table 6:** Results from determination of Irbesartan (IRB), Losartan potassium (LOS) and Telmisartan (TEL) in presence Hydrochlorothiazide (HCTZ) respectively in their dosage forms by reported (published) method

**Product name**	**X-tension** **plus tablets**		**Losazide tablets**		**Micardis plus tablets**	
**Drug name**	**IRB** **(%)**	**HCTZ** **(%)**	**LOS** **(%)**	**HCTZ** **(%)**	**TEL** **(%)**	**HCTZ** **(%)**
Test 1	97.04	97.16	99.82	99.78	100.2	100.1
Test 2	99.44	97.88	99.73	100.6	99.67	99.04
Test 3	99.49	97.38	99.99	99.45	99.53	99.3
Test 4	99.28	99.22	99.97	99.51	99.51	98.56
Test 5	99.67	99.11	100.8	100.5	99.58	98.84
SD	1.10	0.96	0.43	0.55	0.287	0.59
Average	98.98	98.15	100.1	99.97	99.7	99.17
R.S.D	1.11	0.98	0.43	0.550	0.288	0.59
Method number	5		13		11	

**Table 7:** Statistical comparison of the proposed and published methods for determination of Irbesartan (IRB), Losartan potassium (LOS) and Telmisartan (TEL) in presence Hydrochlorothiazide (HCTZ) respectively in their dosage forms by reported method (T- student test) and (F –test for variance).

**Drug name**		**Recovery ± SD**		**Calculated** **t- values**	**Calculated F- values**
		**Proposed methods**	**Reference method**		
X-tension plus tablets	IRB (%)	99.26±0.76	98.98 ±1.10	1.36	0.48
	HCTZ (%)	98.76 ±0.70	98.15 ±0.96	2.14	0.52
Losazide tablets	LOS (%)	100.35±0.56	100.10±0.43	0.84	1.71
	HCTZ (%)	99.47 ±0.50	99.97±0.55	1.15	0.84
Micardis plus tablets	TEL (%)	99.51 ±0.20	99.70±0.287	2.19	0.50
	HCTZ (%)	99.25 ±0.28	99.17±0.59	0.61	0.22

(Where the Tabulated t-values and F -ratios at p = 0.05 are 2.57 and 5.05)

## Conclusion

A simple, accurate, precise, robust and reliable LC method has been established for simultaneous determination for Irbesartan, Losartan potassium, Telmisartan, Valsartan in presence of Hydrochlorothiazide in their formulations. The method has several advantages: The first is using HPLC-UV which is the most available instrument in pharmaceutical analysis in pharmaceutical industrial companies in comparison with methods utilized electrophoresis, HPLC coupling with fluorimetric detection or mass spectroscopy (especially in developing countries). High sensitive method has LOD range (0.005-0.08) μg/mL and LOQ range (0.017-0.24) μg/mL. It is suitable for analysis of antihypertensive agents in their formulations in a single run, in contrast with previous methods. This makes the method suitable for routine analysis in quality-control laboratories. Other merits are rapid analysis, a simple mobile phase, simple sample preparation, does not use polluting reagents. 
